# Cold Signaling and Cold Response in Plants

**DOI:** 10.3390/ijms14035312

**Published:** 2013-03-06

**Authors:** Kenji Miura, Tsuyoshi Furumoto

**Affiliations:** 1 Faculty of Life and Environmental Sciences, University of Tsukuba, Tsukuba 305-8572, Japan; 2 Department of Agriculture, Ryukoku University, Kyoto 610-8577, Japan; E-Mail: tfurumoto@agr.ryukoku.ac.jp

**Keywords:** cold signaling, cold tolerance, ICE1, CBF/DREB1, cold-regulated genes

## Abstract

Plants are constantly exposed to a variety of environmental stresses. Freezing or extremely low temperature constitutes a key factor influencing plant growth, development and crop productivity. Plants have evolved a mechanism to enhance tolerance to freezing during exposure to periods of low, but non-freezing temperatures. This phenomenon is called cold acclimation. During cold acclimation, plants develop several mechanisms to minimize potential damages caused by low temperature. Cold response is highly complex process that involves an array of physiological and biochemical modifications. Furthermore, alterations of the expression patterns of many genes, proteins and metabolites in response to cold stress have been reported. Recent studies demonstrate that post-transcriptional and post-translational regulations play a role in the regulation of cold signaling. In this review article, recent advances in cold stress signaling and tolerance are highlighted.

## 1. Introduction

Only one-third of the total land area on Earth is free of ice and 42% of land experiences temperatures below −20 °C [1]. In such areas, plants require specialized mechanisms to survive exposure to low temperature. Cold stress can be classified as chilling (0–15 °C) and freezing (<0 °C) stresses. Generally, plants originating from temperate regions, such as spinach and *Arabidopsis*, exhibit a variable degree of chilling tolerance and can increase their freezing tolerance during exposure to chilling and non-freezing temperatures. This process is known as cold acclimation [2]. On the other hand, plants of tropical and subtropical origins are sensitive to chilling stress and lack the cold acclimation mechanism.

The molecular basis of cold acclimation and acquired freezing tolerance in plants, mainly *Arabidopsis* and winter cereals, has been extensively studied. To adapt to cold stress during cold acclimation, gene expression is reprogrammed and the metabolism is also modified [3]. Cold response is a very complex trait involving many different metabolic pathways, gene regulations and cell compartments [4]. This review highlights cold stress signaling, the transcriptional and post-translational regulation of gene expression during cold acclimation, the interrelationship between cold responses and plant hormones and the transgenic approach to confer cold tolerance onto plants.

## 2. Cold Stress Sensing and Second Messengers

Thus far, no plant sensors for low temperature have been identified. Multiple primary sensors are thought to be involved in stress sensing. Each sensor may perceive a specific aspect of the stress and may be involved in a distinct branch of the cold signaling pathway. Plants may sense low temperature through changes in the physical properties of membranes, because membrane fluidity is reduced during cold stress [5]. In fact, plasma membrane rigidification raised by a membrane rigidifier, dimethyl sulfoxide (DMSO), can induce the expression of *COR* (cold-responsive) genes, even at normal growth temperatures, whereas the application of a membrane fluidizer, benzyl alcohol, prevents the induction of *COR* gene expression at low temperatures [5,6].

Within seconds of a cold shock, a transient increase in cytosolic Ca^2+^ levels is observed [7]. Membrane rigidification induces cytosolic Ca^2+^ signatures, and the transient increase in Ca^2+^ regulates *COR* gene expression. Because *COR* gene expression was impaired by gadolinium, a mechanosensitive Ca^2+^ channel blocker, it is suggested that mechanosensitive Ca^2+^ channels may be involved in the perception of cold-induced membrane rigidification [7]. The Ca^2+^ signal can be transduced into the nucleus. Nuclear [Ca^2+^], which is monitored by a chimera protein, formed by the fusion of aequorin to nucleaoplasmin, is also transiently increased after cold shock, and the peak of nuclear [Ca^2+^] is delayed at 5 to 10 s, compared to the peak of cytosolic [Ca^2+^] [8]. In animal cells, the increase in nuclear [Ca^2+^] is caused by nuclear envelope, which is continuous with the endoplasmic reticulum, one of major Ca^2+^ stores [9]. The increase in nuclear [Ca^2+^] can be propagated by cytosolic Ca^2+^ transients via the nuclear pore complexes [9]. Because the architecture of nuclear envelope in plants is similar to that described in animal cells with the presence of numerous nuclear pore complexes [10], the nuclear Ca^2+^ signal may be initiated from nuclear envelope and propagated by cytosolic Ca^2+^ transients in plants. Because nuclear Ca^2+^ signaling is also important to control gene transcription in plants [11], as well as animal cells [9], identification of transporters, which are localized to plasma membrane or membrane of nuclear envelope and are involved in regulation of cold-inducible Ca^2+^ transients, may elucidate the detail mechanisms how Ca^2+^ signal regulates cold signaling.

The cold stress-induced Ca^2+^ signature can be decoded by different pathways. Plants possess groups of Ca^2+^ sensors, including CaM (calmodulin) and CMLs (CaM-like), CDPKs (Ca^2+^-dependent protein kinases), CCaMK (Ca^2+^-and Ca^2+^/CaM-dependent protein kinase), CAMTA (CaM-binding transcription activator), CBLs (calcineurin B-like proteins) and CIPKs (CBL-interacting protein kinases). Genetic analysis demonstrated that CDPKs work as positive regulators [12], but calmodulin3 is a negative regulator of gene expression and cold tolerance in plants [13]. CBLs relay the Ca^2+^ signal by interacting with and regulating the family of CIPKs. As the *cbl1* mutant exhibits a chilling sensitive phenotype, CBL1 regulates cold response by interacting with CIPK7 [14]. CAMTA3 has been identified as a positive regulator of *CBF2/DREB1C* expression through binding to a regulatory element (CG-1 element, vCGCGb) in its promoter [15]. The *camta2 camta3* double mutant plants are sensitive to freezing temperatures. The expression of *CBF3/DREB1A* is not regulated by CAMTA, because there is no CG-1 element in its promoter [15].

In addition to the plasma membrane, chloroplast may also play a role in sensing ambient temperature. Under low temperature, an imbalance between the capacity to harvest light energy and the capacity to dissipate this energy through metabolic activity causes excess photosystem II (PSII) excitation pressure, leading to generation of reactive oxygen species (ROS). Detail is described in the section “Chloroplast and cold response”.

The phosphorylation of proteins in response to cold and the suppression of protein phosphatase activity may also provide a means for the plant to sense low temperature. The MPK (mitogen-activated protein kinase) cascade is implicated in the regulation of cold signaling and cold tolerance. *Arabidopsis* MPK4 and MPK6 are phosphorylated by MKK2 (MAP kinase kinase2) when exposed to cold stress, and constitutively activated MKK2 overexpressing plants exhibit cold tolerance and the up-regulation of *CBF/DREB1s* [CRT (C-repeat)/DRE (dehydration responsive-element) binding proteins] [16]. The cold activation of SAMK, an alfalfa MPK, requires membrane rigidification, and the activation of SAMK by low temperatures is inhibited by blocking the influx of extracellular Ca^2+^ and is prevented by an antagonist of CDPKs, suggesting that membrane rigidification, Ca^2+^ fluxes and CDPKs are required for the activation of MPK cascades in alfalfa [17]. Together with these results, several signaling pathways are triggered to promote the production of COR (cold-responsive) proteins.

## 3. ICE-CBF/DREB1 Pathway and Cold-Responsive Gene Regulation

### 3.1. ICEs (Inducer of CBF Expressions) Are Transcription Factors Controlling Cold Signaling through the Regulation of CBF/DREB1s

Among several cold signaling pathways, the *CBF/DREB1*-dependent cold signaling pathway is the best characterized and the key regulatory pathway [18]. In *Arabidopsis*, three CBF/DREB1s are involved in the regulation of *COR* gene expression and cold tolerance [19,20]. The CBF/DREB1 (mainly CBF3/DREB1A) pathway is controlled by a MYC-type transcription factor ICE1 (inducer of *CBF* expression1) [21].

**Figure 1:**
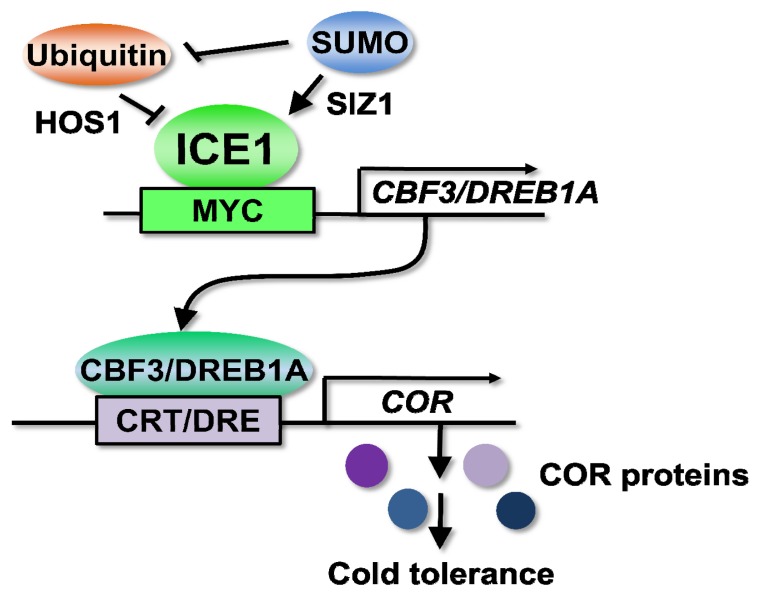
The cold signaling pathway, which involves ICE1 and CBF3/DREB1A. ICE1 is a MYC-type transcription factor and binds to *cis-*elements in the promoter of *CBF3/DREB1A* to induce its expression. CBF3/DREB1A is an AP2-type transcription factor to regulate expression of *COR* (cold-regulated genes) and cold tolerance. Ubiquitylation of ICE1 is mediated by HOS1, an ubiquitin E3 ligase for proteasome-dependent degradation. SIZ1, a SUMO E3 ligase, mediates sumoylation (SUMO conjugation) of ICE1, probably leading to blocking of ubiquitylation and stabilization of ICE1.

The *Arabidopsis ice1* mutant exhibits both chilling and freezing sensitivity, and the overexpression of *ICE1* confers increased freezing tolerance [21]. ICE1 can bind to the MYC recognition *cis*-elements (CANNTG) in the promoter of *CBF3/DREB1A* and induce the expression of *CBF3/DREB1A* and its regulons during cold acclimation (Figure 1) [21]. Approximately 40% of *COR* genes and 46% of cold-regulated transcription factor genes are regulated by *ICE1*, suggesting that ICE1 functions as a master regulator controlling *CBF3/DREB1A* and many other *COR* genes [22]. The *ice1* mutation affects the early cold induction of *CBF1/DREB1B* and *CBF2/DREB1C*, but the induction is not reduced at later times [21], suggesting that other ICE1-like proteins mediate the cold induction of *CBF1/DREB1B* and *CBF2/DREB1C*. Supporting this idea, the overexpression of *ICE2*, a homolog of *ICE1*, enhances the expression of *CBF1/DREB1B* and freezing tolerance in *Arabidopsis*[23]. In the promoter of maize, *ZmDREB1*, the hyperacetylation of histones H3 and H4 and DNA demethylation, occur in the ICE1-binding region after cold treatment, accompanied by chromatin decondensation [24], suggesting that chromatin remodeling is required for the regulation of *CBF/DREB1* by ICE1. Because trichostatin A, an inhibitor of HDACs (histone deacetylases) treatment decreases in induction of *ZmDREB1* and *ZmCOR413*, HDACs may selectively activate transcription [24]. ICE1 is regulated by ubiquitylation [25] and sumoylation (Figure 1) [26] (detail is described in the section “Post-translational regulation”). However, the precise mechanisms for the activation of ICE1 and for transducing signals from perception and the second messengers to ICE1 still remain unknown.

ICE1 is functionally conserved in higher plants (Table 1). The overexpression of *SlICE1* enhances the chilling tolerance and activates the expression of *SlCBF1*, *SlDRCi7* (dehydrin Ci7 homolog) and *SlP5CS* (Δ^1^-pyrroline-5-carboxylase synthase) in tomatoes (*Solanum lycopersicum*) [27]. Furthermore, the accumulation of antioxidants, several amino acids, amines and sugars is increased in red tomato fruits of *SlICE1-*overexpressing plants, leading to an increase in antioxidant activity [28]. Wheat (*Triticum aestivum*) contains two ICE1 homologs, *TaICE141* and *TaICE187*, which regulate the expression of the wheat *CBF* group IV genes. The overexpression of these genes in *Arabidopsis* enhances the *CBF/DREB1-*dependent *COR* gene expression and cold tolerance [29]. *OsICE1* and *OsICE2* in rice (*Oryza sativa*) are induced by cold stress and sequentially upregulate *OsDREB1B, OsHsfA3* (rice heat shock factor A3) and *OsTPP1* (rice trehalose 6-phosphate phosphatase), suggesting that these transcription factors are involved in the response to cold stress [30]. The jasmonate-induced chilling tolerance of the banana plant (*Musa acuminata*) is associated with the induced expression of MaMYC2*,* which interacts with MaICE1 to activate *CBF-*dependent cold signaling [31]. Because the function of ICE1 in the cold response is conserved, the overexpression of *Arabidopsis ICE1* also improves chilling tolerance and enhances the accumulation of soluble sugars and proline in cucumber [32].

### 3.2. The CBF/DREB1 Responsive Pathway

CBF/DREB1s can bind to CRT/DRE *cis-*elements, A/GCCGAC, in the promoter of *COR* genes to regulate expression of *COR* genes [56], and belong to the ERF/AP2 (ethylene-responsive element binding factor/APETALA2)-type transcription factor family [57]. Genomic analyses have revealed that *CBF/DREB1* genes are organized in tandem (*CBF1/DREB1B-CBF3/DREB1A-CBF2/DREB1C*) on *Arabidopsis* chromosome IV [58]. *CBF1/DREB1B* and *CBF3/DREB1A* are induced at the same time and earlier than *CBF2/DREB1C* after cold treatment [59]. Transcriptome analyses in *CBF/DREB1-*overexpressing transgenic plants reveal that approximately 12% of *COR* genes in *Arabidopsis* are controlled by the CBF/DREB1s, but no significant target specificity among the three CBF factors is observed [60,61]. Some transcription factors, such as the ERF/AP2 factors, RAP2.1 and RAP2.6 and the C2H2-type zinc finger, STZ/ZAT10, belong to the CBF-regulon [62,63]. Although no functional difference among three *CBF/DREB1* genes was found in an overexpression analysis, a genetic analysis with the *cbf2* null mutant demonstrates that the freezing tolerance and expression of *CBF1/DREB1B* and *CBF3/DREB1A* are increased in the *cbf2* mutant, suggesting that *CBF2/DREB1C* is a negative regulator of both *CBF1/DREB1B* and *CBF3/DREB1A*[64]. On the other hand, *CBF1/DREB1B* and *CBF3/DREB1A* are not involved in the regulation of other *CBF/DREB1s*, but positively regulate cold acclimation by activating the same subset of CBF/DREB1*-*target genes [65]. These data suggest that CBF1/DREB1B and CBF3/DREB1A have different functions than CBF2/DREB1C. And even though CBF1/DREB1B and CBF3/DREB1A control the same subset of genes, they are concertedly required to induce the whole CBF/DREB1-regulon and complete the development of cold acclimation [18,65].

*CBF/DREB1* homologs have been identified in different species. The functional characterization of these homologs reveals that some have similar functions as have *Arabidopsis CBF/DREB1s*. The overexpression of the homologs from several species in transgenic rice, tobacco or *Arabidopsis* plants enhances the expression of cold-regulated CBF-regulon genes and cold tolerance. Furthermore, growth retardation is observed in these overexpressing plants, as exhibited by *Arabidopsis CBF/DREB1-*overexpressing plants (detail is described in the section “Cold response and plant hormone”) (Table 1) [45–50]. On the other hand, the ectopic expression of barley or *Arabidopsis CBF/DREB1s* in different plants, such as the popular potato, wheat and rice, promotes tolerance to low temperatures (Table 1) [36,40–42]. As for ICE1, the function of CBF/DREB1s seems widely conserved in higher plants. However, as it is noted that no significant increase in cold tolerance is observed in some plants when these factors are overexpressed, differences in CBF/DREB1s function could be considered in different species. The overexpression of either tomato *SlCBF1* or *Arabidopsis AtCBF3* in transgenic tomatoes does not improve cold tolerance, even though these *CBFs* enhance the freezing tolerance of *Arabidopsis*[44]. Additionally, the cold tolerance of transgenic rice expressing *Arabidopsis AtCBF1* is not significantly different from that of wild-type plants [66]; however, the overexpression of a monocot-derived CBF, barley HvCBF4, in rice results in an increase in the tolerance to low temperatures [36], suggesting that the function of barley CBF/DREB1 may be different from that of *Arabidopsis* CBF/DREB1.

A QTL (quantitative trait loci) mapping approach has also demonstrated that *CBFs* play important roles in cold tolerance. Eleven of 20 *CBF/DREB* genes are found in two tight tandem clusters on the long arm of chromosome 5H in the same region as the *Fr-H2* frost tolerance locus in barley [67,68]. In wheat, *Triticum monococcum*, similar *CBF* gene clusters are located at the *Fr-A^m^2* frost tolerance QTL [69,70]. *FpCBF6* in *Festuca pratensis* co-localizes with the major frost tolerance/winter survival QTLs *QFt5F-2/QWs5F-1*[71]. Together, these results indicate that *CBF/DREB* acts a key regulator of cold tolerance in various plants.

### 3.3. Cold-Regulated Genes

The expression of *COR* has been shown to be critical in plants for both chilling tolerance and cold acclimation. *COR78/RD29A, COR47, COR15A* and *COR6.6* in *Arabidopsis* and other plants encode dehydrins, which is known as group 2 LEA (LEA II) proteins [72] and are induced by cold stress [73]. LEA proteins are thought to be important for membrane stabilization and prevent protein aggregation [74]. The cold-inducible dehydrins ERD10 (early response to dehydration10) and ERD14 function as chaperones and interact with phospholipid vesicles through electrostatic forces [75,76]. Several dehydrins, such as wheat WCOR410, barley DHN5, peach PCA60 and citrus CuCOR19, increase tolerance to cold stresses [77–81]. In addition, HSP (heat shock protein) expression is also induced by cold in plants [82]. These HSPs function in membrane protection, in the refolding of denatured proteins and in preventing protein aggregation [82,83]. Some PR (pathogen-related) proteins, such as PR1, PR2 (*β*-1,3-glucanase) and PR5 (thaumatin-like proteins), are induced by cold treatment in *Arabidopsis*[84]. PR10 (Bet v-1 homologues), PR11 (chitinases) and PR14 (lipid transfer proteins) are also cold-inducible in several species [85–92]. The antifreeze activity of *β*-1,3-glucanase, chitinases and thaumatin-like proteins inhibits the recrystallization of intercellular ice in the apoplastic space and prevents intracellular ice formation, as cell dehydration is promoted by extracellular freezing [93]. In addition to these proteins, many enzymes are involved in the cold response machinery, such as detoxification and antioxidant cascades, photosynthesis, lignin metabolism, secondary metabolism, cell wall polysaccharide remodeling, starch metabolism, sterol biosynthesis and oligosaccharide synthesis (reviewed in [93]).

Many studies demonstrate the success of transgenic approaches in increasing tolerance to low temperatures. The chloroplast *GPAT* (glycerol-3-phosphate acyltransferase) of squash, *Cucurbita maxima*, and *Arabidopsis* is involved in phosphatidyl glycerol fatty acid desaturation and increases the ratio of unsaturated fatty acids in plant cell membranes, leading to enhancement of cold tolerance [94]. The citrus LEA gene, *CuCOR19*, enhances the cold tolerance of transgenic tobacco [95]. Similarly, the expression of wheat dehydrin *WCS19*[96], *Arabidopsis COR15A*[97] and the co-expression of *RAB18* and *CO47* or *LTI29/XERO2* and *LTI30/ERD10*[98] increase the freezing tolerance of *Arabidopsis* transgenic plants. The freezing tolerance of transgenic strawberry leaves expressing wheat dehydrin *WCO410* is increased [78]. Rice *TPP1* (trehalose-6-phosphate phosphatase) overexpression enhances the cold tolerance of rice [99]. Because trehalose is a nonreducing disaccharide that functions as a stress protection metabolite, the accumulation of trehalose enhances cold tolerance, as well as other abiotic stresses [100]. These results indicate that *ICE*, *CBF/DREB1* and *COR* genes play important roles in the plant response to low temperatures.

## 4. Post-Transcriptional Regulation

Post-transcriptional mechanisms based on alternative splicing, pre-mRNA processing, RNA stability, RNA silencing and export from the nucleus play critical roles in cold acclimation and cold tolerance. Pre-mRNA processing and export are important processes for the regulation of gene expression in eukaryotes [101]. Plants regulate the stress-dependent export of mRNA from the nucleus and the selective translation of stress-associated genes and increase the stability of related transcripts [102]. Because, at low temperatures, misfolded RNA molecules become over-stabilized, RNA binding proteins function as RNA chaperones that help RNA achieve their native conformation. Glycine-rich protein GRP7 plays a role in the export of mRNA from the nucleus to the cytoplasm under cold stress conditions [103]. The RNA helicase LOS4 (low expression of osmotically responsive gene4) is important for nuclear mRNA export, particularly in response to temperature stress [104]. mRNA export is inhibited by the *los4-1* mutation, leading to the reduced expression of *CBF* and sensitivity to chilling stress. A null mutation in the *AtNUP160* gene, which encodes a nucleoporin protein involved in mRNA export, causes the decreased induction of *CBFs* and some CBF targets in response to cold [105]. These results suggest that mRNA export plays an important role in the regulation of *CBF* expression.

In *Arabidopsis*, the RNA-seq approach revealed that approximately 42% of genes undergo alternative splicing to produce different proteins [106]. EST/cDNA evidence show about 21% of the expressed genes are alternatively spliced in rice [107]. Although most alternative splicing events have not been characterized in plants, several genes encoding protein kinases, transcription factors and splicing regulators have demonstrated the centrality of alternative splicing in the fine-tuning for abiotic stress responses [108]. The pre-mRNA of serine/arginine-rich proteins functions in the regulation of mRNA splicing and alternative splicing of the protein is observed under cold and heat stresses in *Arabidopsis*[109]. CCA1 (circadian clock associated1) and LHY (late elongated hypocotyl) are MYB-type transcription factors that are core components of the circadian clock in *Arabidopsis* and positively affect the expression of *CBF* pathway genes [110]. The *CCA1* transcript is alternatively spliced into two variants: *CCA1α*, the fully spliced variant, and *CCA1β*, which retains the fourth intron. CCA1α-CCA1α, LHY-LHY or CCA1α-LHY function in the regulation of the circadian clock; however, CCA1β, which lacks a DNA binding domain, interferes with the dimerization. Low temperatures suppressed CCA1 alternative splicing; thus, CCA1β is reduced. Because the overexpression of *CCA1α* or *CCA1β* enhances the freezing tolerance or sensitivity, respectively, the alternative splicing of *CCA1* regulated by cold stress contributes to freezing tolerance [111]. *LHY* is also alternatively spliced and the amount of alternatively spliced transcript harboring the long intron of *LHY* is more abundant in cold-adapted plants. Temperature-dependent alternative splicing is important for decreasing the *LHY* transcript abundance upon cooling [112]. The alternative splicing of the *Arabidopsis* transcription factor *IDD14* (indeterminate domain14) produces the IDD14β form, which lacks a DNA-binding domain and is produced predominantly under cold conditions [113]. IDD14β interacts with the functional IDD14α, leading to the reduction of the binding activity of the promoter of the *QQS* (qua-quine starch) gene, which regulates starch accumulation [113]. SR (serine/arginine-rich) proteins are involved in the regulation of mRNA splicing and are also alternatively spliced under cold and heat stresses in *Arabidopsis*[109].

Small non-coding RNAs, namely, micro-RNAs (miRNAs) and small interfering RNAs (siRNAs), play roles as repressors of gene expression in animals and plants [114]. Plant miRNAs are approximately 21 nt-long small regulatory RNAs that are derived from the processing of longer primary miRNA transcripts [115,116]. miRNAs recognize their mRNA targets based on imperfect sequence complementation. The RISC (RNA-induced silencing complex)-containing miRNA/siRNA induces post-transcriptional gene silencing by the cleavage of mRNA and translational repression [117]. Many cold-regulated miRNAs have been identified by cloning, bioinformatics and high-throughput sequencing approaches in *Arabidopsis*[118], *Populus*[119], *Brachypodium*[120] and rice [121]. Although several miRNAs show similarity among species (*miR397* and *miR169* are upregulated), several differences in miRNA regulation have been observed, suggesting specificity among species. So far, little is known about the miRNA target genes, whose expression levels are altered in response to cold. The functional analysis of *miR169* for cold response has not been characterized, but it is reported that *miR169* is involved in the regulation of nitrogen-starvation responses. The *miR169a* overexpressing plants accumulate less nitrogen and are more sensitive to nitrogen starvation stresses than wild-type [122]. No phenotypic analysis of *miR397* has been performed. Cu/Zn SODs (superoxide dismutases) are targets of *miR398*, whose expression level is downregulated by cold and oxidative stress in *Arabidopsis*[123]. The stress-induced reduction in *miR398* expression results in the accumulation of Cu/Zn SOD transcripts, suggesting that *miR398* plays a role in the regulation of ROS detoxification under abiotic stresses.

## 5. Post-Translational Regulation

The ubiquitylation of a protein leads to its degradation by the 26S proteasome. The ubiquitin-proteasome pathway plays important roles in many biological functions, including abiotic stress responses [124]. *Arabidopsis* HOS1 (high expression of osmotically responsive gene1) is an ubiquitin E3 ligase that exerts a negative control on cold response and degrades ICE1 (Figure 1) [25]. The *hos1* mutant exhibits upregulation of *CBF/DREB1s* and several cold-regulated genes with cold treatment [125]. HOS1 is shuttled from the cytoplasm to the nucleus during cold acclimation for the poly-ubiquitylation of ICE1 [126]. The substitution of serine 403 of ICE1 to alanine promotes the stabilization of ICE1. The ubiquitylation of ICE1(S403A) is inhibited, and the overexpression of ICE1(S403A) enhances cold tolerance more than the overexpression of wild-type ICE1 [127]. A recent study has demonstrated that the *hos1* mutant also exhibits early flowering only under short day conditions, and this photoperiodic control of flowering is caused by HOS1 negatively regulating the levels of the CO (constans) protein, particularly during the daytime [128]. HOS1 is also subjected to alternative splicing, and the overexpression of *HOS1-L* delays flowering, compared to that of *HOS1-S*[129]. On the other hand, HOS1 controls flowering time in response to low ambient temperatures (16 °C) and intermittent cold, most likely through the interaction between HOS1 and FVE or FLK (flowering locus K) [130]. Another study demonstrates that intermittent cold treatment triggers the degradation of CO by HOS1, suggesting that the HOS1-CO module contributes to the fine tuning of photoperiodic flowering under short-term temperature fluctuations [131].

The overexpression of another E3 ligase, AtCHIP (carboxyl terminus of HSC70-interacting protein), causes increased sensitivity to low temperatures compared with the wild-type, suggesting its role as a negative regulator of cold response [132]. In wheat, *Triticum durum*, the RING-finger E3 ligase TdRF1 (RING-finger protein1) interacts with another RING-finger E3 ligase, WVIP2 (wheat viviparus1 interacting protein2), and both genes are upregulated by cold treatment [133]. The transcription factor, WBLH1 (wheat Bel1-type homeodomain1), is degraded in a TdRF1-dependent manner through the 26S proteasome [133]. The overexpression of *TdRF1* increases the tolerance of barley cells to dehydration, suggesting that TdRF1 may function in the protection of plants from drought and freezing conditions.

SUMO (small ubiquitin-related modifier) conjugation, or sumoylation, is one of the post-translational modifications [134,135]. Sumoylation plays an important role in a wide variety of cellular processes, including changes in enzyme activity, the regulation of transcription- and chromatin-related processes, sub-cellular relocalization and protection from the ubiquitin-mediated degradation of regulatory proteins [136]. Similar to the ubiquitylation system, sumoylation is processed through the SUMO-specific E1, E2 and E3 enzymes, and SUMO is covalently conjugated to the target consensus motif ΨKxE/D (Ψ, hydrophobic amino acid; K, SUMO target lysine; x, any amino acid; E/D, glutamic acid/aspartic acid). Both loss- and gain-of-function analyses reveal that sumoylation functions in the regulation of responses to abiotic and biotic stresses [134,135,137], such as the response to nutrient availability [138–140], drought tolerance [141,142], basal thermotolerance [143], salt stress tolerance [144,145], copper tolerance [146] and innate immunity [147,148], as well as the development [149–153] and regulation of ABA signaling [154,155]. As the *siz1* mutant, which is impaired in the SUMO E3 ligase [138], exhibits hypersensitivity to chilling and freezing stresses [26], sumoylation contributes to the regulation of cold signaling through the stabilization of ICE1. SUMO conjugation to ICE1 blocks its ubiquitylation, allowing for the activation of *CBF3/DREB1A* transcription (Figure 1). Recent studies have identified hundreds of potential targets for sumoylation [156–159]. Because several transcription factors and chromatin-modifying factors are potential sumoylated targets, these proteins may be involved in the regulation of cold signaling and tolerance. Some examples are GCN5, a histone acetyltransferase and STA1 (stabilized1). GCN5 is an interacting protein of CBF1 and ADA2 [160]. A transcriptional adaptor and the transcription of *COR* genes was reduced in the *gcn5* mutant [161]. STA1 is a pre-mRNA splicing factor that catalyzes the splicing of *COR15A* for cold tolerance [162].

## 6. Cold Response and Plant Hormones

Low temperature affects several aspects of plant adaptation, e.g., freezing tolerance, plant growth, abiotic resistance and senescence [163]. Among phytohormones, ABA, auxin, gibberellic acid (GA), salicylic acid (SA) and ethylene are related to the cold responses positively or negatively. Here, the effects of these phytohormones on the cold response are summarized.

The ABA level increases in response to low temperatures [164,165]. The *CBF* genes are also induced by exogenous ABA [166]. Among cold responses, ABA is not related to vernalization, as an exogenous ABA treatment is not able to induce the expression of a vernalization marker gene, *VIN3*, but induces the expression of another cold-regulated gene, *ADH1*[167]. These observations are consistent with early reports showing that the cold signaling associated with vernalization is unnecessary for ABA [168,169] and indicates that the limited effect of ABA to cold-responsive transcriptional regulation.

Temperature has strong effects on the architecture of some plants. Among the above hormones, auxin and GA are directly related to cell-elongation under warm conditions [170]. On the other hand, in the case of *Arabidopsis*, cold treatment upregulates the *GA 2-oxidase* gene, encoding the GA-catabolizing enzyme, and represses the *GA 20-oxidase* gene, encoding a GA-biosynthesizing enzyme. A wheat variety, reduced height 3 (Rht3), exhibits a dwarf phenotype under 20 °C, but normal growth under 10 °C. Rht3 is defective in GA signaling, indicating the importance of GA signaling for warm temperature-dependent cell growth [171]. Hence, the regulation of the GA level is a key output of temperature signaling for determining plant architecture. Under cold or cool conditions, endogenous GA and auxin levels are thus reduced, leading to dwarf architecture in Rht3 wheat variety. The growth retardation of *CBF1/DREB1B-*overexpressing plants is caused, in part, by accumulation of DELLA proteins [172]. DELLA is degraded by proteasome and degradation of DELLA is enhanced by GA [173]. Thus, growth inhibition of *CBF1/DREB1B-*overexpressing plants is suppressed by the application of GA and the introduction of the double DELLA mutation *gai-t6 rga-24*[172]. This data indicates that CBF/DREB1-dependent signaling pathway regulates plant growth through modulation of DELLA protein accumulation.

Endogenous free SA and glucosyl SA are accumulated during chilling in *Arabidopsis* shoots, wheat and grape berry [174–176]. And SA treatment enhances the cold tolerance of various spices, such as rice, maize, wheat, potato, *etc*. [177–180]. However, high concentrations and continual application of SA cause growth retardation. Several *Arabidopsis* mutants, such as *cpr1* (constitutive expresser of pathogenesis-related gene1) and *acd6* (accelerated cell death6), in which SA is over-accumulated, exhibit a dwarf phenotype and freezing sensitivity [175,181]. ICE1 is a transcription factor classified as a member of the basic Helix-Loop-Helix type transcription factors that binds the promoter of *CBF3/DREB1A* and, therefore, is one of the central regulators of cold signaling, as described above [163]. In addition, in the *ice1* mutant, SA-inducible genes are upregulated [21]. These results indicate the essential function of ICE1 as a cold- and SA-signaling integrator. CAMTA3/AtSR1, a member of the calmodulin binding transcription activator family, recognizes the promoter of *CBF2/DREB1C* to positively regulate cold tolerance [15] and the promoter of *EDS1* to repress disease resistance [182]. These results suggest that cold signaling and SA signaling are interrelated.

Leaf senescence is a complex and tightly regulated process related to cold stress [183]. *CBF2/DREB1C* overexpression in *Arabidopsis* shows a delay of senescence in both natural and artificially induced senescence conditions. In the latter cases, an extended lifespan was observed under the darkness, ethylene or ABA treatments, although the detailed molecular mechanism still remains to be solved [184].

## 7. Chloroplast and Cold Response

As described above, low temperature affects plant architecture. How cold temperatures are sensed is the great enigma for plant scientists. One hypothesis is that the energy balance in chloroplasts may function in sensing ambient temperature [185,186]. Although enzymatic activities are usually limited and dependent on low temperature, the light harvesting system is not affected by the ambient temperature. At low temperature, an imbalance between the capacity for harvesting light energy and the capacity to consume this energy on metabolic activity occurs in leaves. It causes an excess PSII excitation pressure, leading to either reversible downregulation of PSII through the dissipation of excess absorbed energy or the irreversible inactivation of PSII and damage to the D1 reaction center proteins and subsequent inhibition of the photosynthetic capacity [187]. The process is called photoinhibition. Because of the over-reduction of PSII, this imbalance might generate reactive oxygen species (ROS), which cause destruction of the photosynthetic apparatus and damage of whole cells and which presumably function as the second messenger [188,189]. Because the photosynthetic fixation of CO2 is very limited under low temperatures, photoinhibition occurs even under relatively low irradiance [190]. Thus, tolerance to the cold-induced photoinhibition seems to be a mechanism for cold acclimation and to be closely related to freezing tolerance. Over-reduction of PSII may also act as one of the signals triggering gene expression of cold regulated genes in rye, because several cold-inducible genes are also induced by high irradiation [191]. In addition, cold-tolerant crop species, such as spinach, winter wheat, rye and faba bean, are able to maintain the high CO_2_ assimilation rate, due to changing RubisCO content and nitrogen efficiency, depending on the ambient temperature, whereas cold-sensitive species, such as cucumber, tobacco and rice, are not [192]. Light strength and ambient temperature may be integrated into the alteration of the electron sink in chloroplasts and initiate a signal transduction pathway [186], explaining the coordination of gene expression between plastids and nuclei, which is necessary for proper adaptation to ambient temperature. Various strategies and mechanisms are suggested for acclimation of the photosynthetic apparatus to low temperatures. One of strategies is an increase in the RuBP-regeneration, enhancing the electron flux through the Calvin cycle and photosynthetic capacity in cereals [193–195]. Another strategy is non-photochemical quenching and thermal deactivation of excess light energy occurring within the PSII reaction center (reaction center quenching) [196,197]. *Arabidopsis* can modify the state transition, PSII and PSI balance, through the phosphorylation and migration of LHCII as cold responses [198]. Despite much physiological evidence supporting this hypothesis, there is little information at the genetic and/or molecular levels, and the molecular mechanisms remain to be elucidated.

## 8. Conclusions and Perspective

The complex and interactive relationships among different pathways are involved in the regulation of cold acclimation. Among them, the ICE1-CBF/DREB1-dependent pathway is likely to play a central role in regulation of cold signaling, as described above (Figure 1). ICE1 and CBF/DREB1 are also conserved and function in an important role in the regulation of cold signaling in many plants (Table 1). Furthermore, several post-transcriptional and post-translational modifications have been identified. Ubiquitylation and sumoylation are involved in the control of cold signaling through regulation of ICE1. As alternative splicing of clock genes is a regulator for cold signaling, the alternative splicing mechanism may be important to integrate clock and cold responses. Cold signaling and cold responses seem to be regulated by several factors, such as Ca^2+^ signaling and chloroplast status, as well as clock genes. Identification of CAMTA is direct evidence to connect between Ca^2+^ signaling and regulation of *CBF/DREB1*. Because the Ca^2+^ concentration is transiently increased by cold shock in the cytosol and nucleus, decoding of this Ca^2+^ signature is required for transducing cold signaling downstream. CAMTA may be one of the candidates for a decoder. The next big challenge should be identification of cold sensors. As described above, multiple primary sensors are thought to be involved in sensing low temperatures, and each sensor may perceive a specific aspect of the stress. Perception of membrane rigidification and an imbalance between capacity of light-energy harvest and of energy dissipation may be included. The elucidation of the sensory mechanism and signaling mechanism from sensor(s) to cold signaling is an important aim in achieving a complete understanding of the cold signaling mechanism.

## Figures and Tables

**Table 1:** ICE1-CBF/DREB1-dependent signaling components conferring plant cold tolerance.

Gene	Transgenic host	Source plant	Phenotype and effects	References
AtICE1	*Arabidopsis thaliana*	*Arabidopsis thaliana*	Freezing tolerance; activation of CBF3/DREB1A	[21]
AtICE2	*Arabidopsis thaliana*	*Arabidopsis thaliana*	Freezing tolerance; activation of CBF1/DREB1B	[23]
AtICE1	*Cucumis sativus*	*Arabidopsis thaliana*	Chilling tolerance; dwarf	[32]
SlICE1	*Solanum lycopersicum*	*Solanum lycopersicum*	Chilling tolerance; accumulation of antioxidants	[27,28]
TaICE141, TaICE187	*Arabidopsis thaliana*	*Triticum aestivum*	Freezing tolerance	[29]
AtCBF1, AtCBF2, AtCBF3	*Arabidopsis thaliana*	*Arabidopsis thaliana*	Freezing, salt and drought tolerance; constitutive expression of *COR*	[20,33,34]
OsDREB1A, OsDREB1B, OsDREB1C	*Oryza sativa*	*Oryza sativa*	Chilling, salt and drought tolerance; dwarf	[35]
HvCBF4	*Oryza sativa*	*Hordeum vulgare*	Chilling, drought and salt tolerance	[36]
TaDREB2, TaDREB3	*Triticum aestivum*	*Triticum aestivum*	Freezing and drought tolerance; dwarf	[37]
AtCBF1, AtCBF2, AtCBF3	*Brassica napus*	*Arabidopsis thaliana*	Freezing tolerance; constitutive expression of *COR*	[38]
AtCBF1	*Fragaria ananassa*	*Arabidopsis thaliana*	Freezing tolerance	[39]
AtCBF3	*Solanum tuberosum*	*Arabidopsis thaliana*	Freezing tolerance	[40]
AtCBF1	*Populus tremula x alba*	*Arabidopsis thaliana*	Freezing tolerance	[41]
AtCBF3	*Triticum aestivum*	*Arabidopsis thaliana*	Freezing tolerance	[42]
AtCBF3	*Nicotiana tabacum*	*Arabidopsis thaliana*	Freezing tolerance	[43]
SlCBF1	*Arabidopsis thaliana*	*Solanum lycopersicum*	Freezing tolerance	[44]
OsDREB1A	*Arabidopsis thaliana*	*Oryza sativa*	Freezing, drought and salt tolerance	[45]
ZmDREB1A	*Arabidopsis thaliana*	*Zea mays*	Freezing and drought tolerance; dwarf	[46]
VrCBF1, VrCBF4	*Arabidopsis thaliana*	*Vitis riparia*	Freezing and drought tolerance; dwarf	[47]
HvCBF3	*Arabidopsis thaliana*	*Hordeum vulgare*	Freezing tolerance	[48]
LpCBF3	*Arabidopsis thaliana*	*Lolium perenne*	Freezing tolerance; dwarf	[49,50]
SlCBF1	*Arabidopsis thaliana*	*Solanum lycopersicum*	Chilling and oxidative tolerance	[51]
MbDREB1	*Arabidopsis thaliana*	*Malus baccata*	Chilling, drought and salt tolerance	[52]
GmDREB3	*Arabidopsis thaliana*	*Glycine max*	Freezing, drought and salt tolerance	[53]
BpCBF1	*Arabidopsis thaliana*	*Betula pendula*	Freezing tolerance; dwarf	[54]
OsDREB1B	*Nicotiana plumbaginifolia*	*Oryza sativa*	Freezing, oxidative and drought tolerance; disease resistance	[55]
